# International Pediatric Emergency Medicine and Critical Care Fellow Education: Utilizing Virtual Resuscitation Simulation in Settings With Differing Resources

**DOI:** 10.7759/cureus.21991

**Published:** 2022-02-07

**Authors:** Christine E Maloney, Rebekah Burns, Emily Hartford, Amelie von Saint Andre-von Arnim, Sarah Foohey, Mukokinya Kailemia, Bhupi Reel, Anita Thomas

**Affiliations:** 1 Pediatrics, Seattle Children's Hospital, Seattle, USA; 2 Pediatric Emergency Medicine, Seattle Children's Hospital, Seattle, USA; 3 Critical Care Medicine, Seattle Children's Hospital, Seattle, USA; 4 Family and Community Medicine, University of Toronto, Toronto, CAN; 5 Paediatric Intensivist, University of Nairobi, Nairobi, KEN

**Keywords:** fellow education, pediatric shock, resuscitation and simulation research in pediatrics, virtual learning, global health education

## Abstract

Pediatric Emergency and Critical Care-Kenya (PECC-Kenya) is an international collaboration between the University of Nairobi and the University of Washington (UW) supporting a combined fellowship program in pediatric emergency medicine (PEM) and pediatric critical care medicine (PCCM) in Kenya. Typically, PEM/PCCM faculty from UW travel to Kenya to support in-person simulation, which was cancelled due to COVID-19 travel restrictions. This presented a need for alternative modalities to continue simulation-based education. This technical report describes the use of virtual simulation for pediatric emergency and critical care fellow education on the management of hypovolemic and septic shock, utilizing international guidelines and being based on resource availability.

## Introduction

Simulation-based education has become an integral part of medical education in high-income countries, particularly in the field of emergency medicine, where providers are required to be proficient in the management of a wide variety of disease pathologies and procedural skills [1]. However, traditional simulation training often relies on dedicated simulation centers and advanced technology and can be resource-intensive. Virtual simulation training, also known as telesimulation, remote or distance simulation, uses the internet to connect educators and learners in remote locations [2]. This offers advantages including low-cost, ease of customization, and wide accessibility. Virtual simulation training has been shown to yield similar educational outcomes when compared with in-person training [3-4]. Given these advantages, virtual simulation is an optimal tool for use in rural or remote settings or settings without ready access to a simulation center.

Utilization of virtual simulation presented an opportunity to continue international medical simulation education amidst the COVID-19 pandemic with travel restrictions and limits to in-person educational activities. To continue supporting the education of pediatric emergency medicine (PEM) and pediatric critical care medicine (PCCM) fellows in Kenya, the existing international collaboration between faculty from the University of Washington and the University of Nairobi developed three virtual simulation scenarios. The virtual simulation scenarios were developed with a focus on international management of pediatric shock, using an existing virtual simulation platform called Virtual Resus Room, developed by Dr. Sarah Foohey [5].

Globally, diarrheal illness and pneumonia, with progression to circulatory instability, are leading causes of pediatric mortality [6]. Patients presenting with shock and circulatory impairment traditionally receive fluid resuscitation as part of their initial management. However, over the last 10 years, studies have raised caution about fluid resuscitation for patients presenting with shock depending on the available resources [7-8]. It is critical for providers around the world to understand the differing international guidelines.

The World Health Organization (WHO) requires three clinical criteria to be met to determine that a patient is in shock, including “cold extremities with capillary refill time greater than 3 seconds and a weak and fast pulse” [8]. Notably, this definition allows for providers to quickly identify a patient in shock in settings where a blood pressure cuff may not be readily available.

Fluid resuscitation recommendations for patients in shock differ depending on the guideline. This is largely due to evidence from a large randomized trial of children with fever and impaired perfusion in East Africa, where mortality was increased in those who received crystalloid volume expansion [7]. The WHO Emergency Triage Assessment and Treatment (ETAT) guidelines recommend that patients presenting with shock, using the clinical definition described above, without severe anemia or malnutrition, receive a volume of 10-20 cc/kg of crystalloid fluid run over 30-60 minutes followed by a second bolus, if the patient did not have clinical improvement, of 10 cc/kg run over 30 minutes [8]. If a patient does not meet all three clinical criteria for shock per the WHO definition, it is recommended that fluid boluses be avoided and that children instead receive maintenance fluids. In contrast, the Surviving Sepsis International Guidelines, from the Society of Critical Care Medicine, for settings where there is ready access to an intensive care unit, recommend 40-60 cc/kg of crystalloid fluid rapidly within the first hour of care [9]. If there is no access to an intensive care unit, the Surviving Sepsis Guidelines recommend using blood pressure as a decision point. If the patient is hypotensive, it is recommended to give up to 40 cc/kg of fluid within the first hour. If the patient is not hypotensive, maintenance fluids are recommended and fluid boluses should be avoided.

Prompt recognition of a patient in shock, as well as a strong foundational knowledge of the global resuscitation guidelines, is crucial to reduce the morbidity and mortality from these common childhood illnesses in any setting. The goal of these case materials was to provide simulation education resources for providers practicing in low- to middle-income countries utilizing a virtual platform to increase international accessibility.

## Technical report

Introduction

This set of simulations was designed to highlight the differences in shock management using international guidelines and prompt discussion and awareness about how availability of hospital resources can change recommended management. The scenarios were developed in collaboration between the University of Washington and University of Nairobi faculty. These simulations address the most common etiologies of shock globally including hypovolemic shock (Cases 1 and 2) and septic shock (Case 3), as well as common complications including hypoglycemia, electrolyte derangement, and volume overload. These simulations were designed using an existing, open-access, Google Slides (Mountain View, CA) based virtual simulation platform, VRR, with a video conferencing platform [5].

Participants

There were two target audiences for these simulations. The primary target audience was pediatric critical care and emergency medicine fellows practicing in low- to middle-income countries. The secondary audience was pediatric critical care or emergency medicine residents, fellows, or faculty traveling from high-income countries to low- to middle-income countries. For the secondary audience, simulations could be used prior to travel to prepare physicians to manage shock in settings with variable resources using international guidelines.

Setting and equipment

The simulations were designed to be entirely virtual to promote accessibility and international collaboration. Participants were required to have a computer and internet connection. For each case scenario, a Google Slides deck was developed using the free template materials available online at VRR [5]. The slide decks were shared with participants and facilitators at the time of the simulation, and participants interacted and edited the slides simultaneously throughout the case scenarios. The slides were designed to be customizable to allow for modification of the equipment and medications depending on resource availability.

Scenario template

Facilitator guides were developed for each of the three case scenarios (Tables 1-3) following a deteriorating patient scenario framework [10]. Each guide outlines the primary learning objectives, critical actions, case branch points, anticipated flow, and anticipated mistakes for each of the three scenarios. The cases were designed to increase difficulty and medical decision-making to allow learners time to adapt to the simulation platform.

**Table 1:** Case 1 Facilitator Guide HEENT, head, ears, eyes, nose, throat; HPI, history of present illness; GU, genitourinary

Case 1 Template	
Patient information	Name: Joseph; age: 3 years; weight: 14 kg; chief complaint: diarrhea, lethargy
Brief narrative description of case	Joseph is presenting with his mother to a rural clinic in the setting of seven days of non-bloody diarrhea. He presents with clinical evidence of hypovolemic shock. Anticipated interventions include fluid boluses and empiric dextrose at the rural hospital with minimal improvement. It is recommended that he is transferred to a referral hospital for additional management.
Primary learning objectives	Identify a patient in shock relying on the WHO clinical definition
	Differentiate the etiology of shock from the patient’s clinical presentation
	Identify appropriate initial therapy for a patient presenting with hypovolemic shock
	Recognize when referral to a hospital with additional resources is indicated
	Gain experience with the virtual simulation format
Critical actions	Perform initial primary survey at the start of the simulation case
	Identify that the patient meets the clinical definition of shock (cold extremities, capillary refill time > 3 seconds, weak, and fast pulse)
	Assess the child for degree of dehydration and for signs of malnourishment
	Obtain IV access
	Initiate bolus of normal saline or lactated ringers per WHO shock guidelines
	Empirically administer glucose
	Evaluate for clinical improvement and recommend referral to district-level hospital where additional resources are available
Learner preparation	World Health Organization Emergency Triage Assessment and Treatment guidelines
	Virtual Resus Room participant guidelines (https://virtualresusroom.com/761-2/)

Initial presentation	
Initial vital signs	Temperature 37.2°C
	Pulse rate 160 beats per minute
	Respiratory rate 40 breaths per minute
	Blood pressure cuff unavailable
Overall appearance	Patient is lying in mother’s arms and appears lethargic
Actors and roles in the room at case start	Doctor #1: team leader
	Doctor #2: code recorder
	Nurse #1: medication administration
	Observer #1 (if available): critical actions checklist
	Observer #2 (if available): communication observer
	Facilitator #1: verbal simulation facilitator
	Facilitator #2: slide simulation facilitator
	Facilitator #3 (if available): parent
HPI	Mother describes that for the last seven days Joseph has had profuse watery diarrhea and decreased oral intake. Several of the other children in the home have had similar symptoms. Today, when mom tried to wake him up, he appeared sleepier than usual, which is what prompted their presentation to the clinic. If asked for review of systems: mom does not think he has had a fever. No vomiting or apparent abdominal pain. No blood in his stools. Mom does not recall the last time he urinated. No nasal congestion or cough. If asked if he has received any medications: mom gave a traditional medication yesterday (she is unsure of the name) that she obtained from their local dispensary. No other medications.
Past medical/surgical history	Previously healthy
Medications/allergies/immunizations	No daily medications; no known allergies; up to date on immunizations
Family history	No family history
Social history	Lives with mother, father, and three older siblings
Physical Examination	
General	Lying in mother’s arms, lethargic. No apparent distress. Child appears well-nourished.
HEENT	Eyes are closed and appear sunken. Lips are cracked. Mucous membranes dry.
Neck	Supple. No lymphadenopathy.
Lungs	Tachypnea. Lungs clear to auscultation bilaterally. No wheezes, rales, or rhonchi. No retractions. No nasal flaring.
Cardiovascular	Tachycardic. Normal S1 and S2. No murmurs, rubs, or gallops. Capillary refill 4 seconds. 1+ radial pulses bilaterally. No peripheral edema.
Abdomen	Abdomen is soft and non-tender. No rebound or guarding. Bowel sounds active.
Neurological	Eyes are closed though open to voice and stimuli then falls back asleep. Moves all extremities.
Skin	Poor skin turgor. Extremities are cold to touch. Pale. No rash.
GU	Testes descended bilaterally.

Instructor Notes - Changes and Case Branch Points		
Intervention/Time Point	Change in Case	Additional Information
Perform primary survey to assess airway, breath sounds, and circulation	Vital signs: temperature of 37.2°C, heart rate of 160 beats per min, respiratory rate of 40 breaths per min, blood pressure cuff is unavailable	Given patient’s circulatory examination, he meets the clinical definition of shock
	Visual assessment: the patient appears lethargic lying in mother’s arms, he is somnolent but arousable to painful stimuli	
	Airway: intact, patient moaning	
	Breath sounds: lungs are clear to auscultation, tachypneic, no retractions	
	Circulation: cold extremities, capillary refill time 6 seconds, tachycardic, weak pulse	
History is gathered from the mother to determine the likely etiology of shock presentation	Mom describes history of profuse watery diarrhea x 7 days and poor oral intake	Team leader states that history is most consistent with hypovolemic shock
Patient is assessed for malnutrition	No visible wasting, no peripheral edema	
IV access is requested	IV placed	
20 cc/kg normal saline or lactated ringer bolus is hung to be run over 30 minutes	No immediate change	
Point-of-care electrolytes or glucose requested	No change	Facilitator states that they are unable to obtain labs at this facility
Glucose is administered empirically (5 cc/kg of 10% glucose solution infused rapidly by IV injection)	Patient begins to appear awake; eyes are open and looking around the room	
Repeat assessment after the first fluid bolus is administered	Vital signs: heart r of 150 beats per min, respiratory rate of 36 breaths per min	Team leader states that the patient has had minimal improvement in his circulatory status
	Circulation: extremities remain cold, capillary refill time of 4 seconds, tachycardic, weak pulse	
Second 10 cc/kg normal saline or lactated ringer bolus hung to run over 30 minutes	No immediate change	If a larger volume bolus or faster rate of administration is used, the patient develops tachypnea, and facilitator recommends urgent transfer to referral hospital for additional respiratory support
Repeat assessment after the second bolus is administered	Vital signs: heart rate of 140 beats per min, respiratory rate of 32 breaths per min	Team leader recommends transfer to referral or district-level hospital for ongoing management
	Circulation: extremities remain cold, capillary refill time 3-4 seconds, tachycardic, pulses stronger	

Anticipated Mistakes	
Mistake	Tips for Management
Failure to recognize that the patient is in shock	Some learners rely on the patient’s blood pressure to guide the diagnosis of shock, facilitator can prompt “what other clinical signs can we use to assess this patient’s circulatory status”
Failure to empirically give dextrose	When point-of-care glucose testing is unavailable, it is recommended that patients presenting with hypovolemic shock empirically receive IV glucose
Failure to recognize that transfer to a referral facility is indicated	Due to the risk of significant electrolyte abnormalities with hypovolemic shock, as well as risk of volume overload with multiple fluid boluses, referral to a hospital with higher levels of care should be considered

**Table 2:** Case 2 Facilitator Guide HEENT, head, ears, eyes, nose, throat; HPI, history of present illness; GU, genitourinary; CPR, cardiopulmonary resuscitation

Case 2 Template	
Patient information	Name: Stephen; age: 2 years old; weight: 12 kg; chief complaint: diarrhea, lethargy
Brief narrative description of case	Stephen is presenting with eight days of profuse watery diarrhea to a district hospital. His presentation is consistent with hypovolemic shock. Anticipated initial management includes fluid resuscitation and emergency labs, which reveal electrolyte abnormalities including hypernatremia and hypokalemia. If the electrolyte derangements are not corrected, the patient goes into pulseless ventricular tachycardia requiring CPR and defibrillation.
Primary learning objectives	Identify a patient in shock relying on the WHO clinical definition
	Differentiate the etiology of shock from the patient’s clinical presentation
	Identify appropriate initial therapy for a patient presenting with hypovolemic shock
	Manage common electrolyte derangements seen in hypovolemic shock
	Manage pulseless ventricular tachycardia
Critical actions	Perform initial primary survey at the start of the simulation case
	Identify that the patient meets the clinical definition of shock (cold extremities, capillary refill time > 3 seconds, weak, and fast pulse)
	Assess the child for degree of dehydration and for signs of malnourishment
	Obtain IV access
	Initiate bolus of normal saline or lactated ringers per WHO shock guidelines
	Obtain and interpret emergency labs including electrolytes and glucose
	Obtain and interpret ECG
	Initiate appropriate treatment for hypokalemia
	Initiate appropriate interventions for pulseless ventricular tachycardia
Learner Preparation	World Health Organization Emergency Triage Assessment and Treatment Guidelines
	Virtual Resus Room participant guidelines (https://virtualresusroom.com/761-2/)

Initial Presentation	
Initial vital signs	Temperature: 37.4°C
	Heart rate: 180 beats per min
	Respiratory rate: 42 breaths per min
	Blood pressure cuff is unavailable
Overall appearance	Patient appears lethargic on the stretcher but arousable
Actors and roles in the room at case start	Doctor #1: team leader
	Doctor #2: code recorder
	Nurse #1: medication administration
	Observer #1 (if available): critical actions checklist
	Observer #2 (if available): communication observer
	Facilitator #1: verbal simulation facilitator
	Facilitator #2: slide simulation facilitator
	Facilitator #3 (if available): parent
HPI	Father describes that for the last eight days Stephen has had profuse watery diarrhea. He has appeared sleepy over the last two days. He has had three episodes of vomiting. If asked for review of systems: no fever. No apparent abdominal pain. No blood in his vomit or stools. He has urinated once today. He has had decreased appetite and fluid intake.
Past medical/surgical history	Previously healthy
Medications/allergies/immunizations	No daily medications; no known allergies; up to date on immunizations
Family history	No family history
Social history	Lives with parents
Physical Examination	
General	Lying on stretcher. Appears well nourished. Intermittently moaning though no apparent distress.
HEENT	Eyes are close. Mucous membranes are dry. Oropharynx is unremarkable.
Neck	Supple. No lymphadenopathy.
Lungs	Lungs clear to auscultation bilaterally. No wheezes, rales, or rhonchi. No retractions. No nasal flaring.
Cardiovascular	Tachycardic. Normal S1 and S2. No murmurs, rubs, or gallops. Capillary refill 4 seconds. No edema.
Abdomen	Abdomen is soft and non-tender. No rebound or guarding. Bowel sounds active.
Neurological	Eyes are closed though open to voice and stimuli. Moves all extremities.
Skin	Extremities are cold to touch. Pale. No rash.
GU	Normal external male genitalia, testes descended bilaterally.

Instructor Notes - Changes and Case Branch Points		
Intervention/Time Point	Change in Case	Additional Information
Perform primary survey to assess airway, breath sounds, and circulation	Vital signs: temperature of 37.4°C, heart rate of 180 beats per min, respiratory rate of 42 breaths per min, O_2_ saturation of 98%, blood pressure cuff is unavailable	Given patient’s circulatory examination, he meets the clinical definition of shock
	Visual assessment: the patient appears lethargic on stretcher, he opens eyes to voice	
	Airway: intact, patient moaning	
	Breath sounds: lungs are clear to auscultation, tachypneic, no retractions	
	Circulation: cold extremities, capillary refill time > 4 seconds, tachycardic, weak pulse	
History is gathered from the parent to determine the likely etiology of shock presentation	Father describes history of profuse watery diarrhea x 8 days and poor oral intake	Team leader states that history is most consistent with hypovolemic shock
Patient is assessed for malnutrition	No visible wasting, no peripheral edema	
IV access is requested	IV placed	
20 cc/kg normal saline or lactated ringer bolus is hung to be run over 30 minutes	No immediate change	Facilitator should ask “how would you like to give this”
Electrolytes and glucose requested	Patient awakens and appears in pain, unable to localize	Labs announced one minute later
		Sodium: 154 mEq/L
		Potassium: 1.8 mEql/L
		Chloride: 110 mEq/L
		Bicarbonate: 11 mEq/L
		Glucose: 40 mg/dL (2.2 mmol/L)
ECG is requested	ECG: reveals T wave flattening and visible U wave	If ECG changes are not recognized, facilitator should ask about the ECG: “That ECG doesn’t look normal - aren’t there supposed to be T waves?”
Glucose is administered (5 cc/kg of 10% glucose solution infused rapidly by IV injection)	Patient is slightly more awake, interactive	
IV potassium chloride is requested to add to maintenance fluids (40 mEq)	No change	If potassium chloride is requested and hypokalemia is corrected, skip the following 3 rows. If unrecognized, the patient becomes unresponsive with pulseless ventricular tachycardia
Participants recognize pulseless ventricular tachycardia	Patient is unresponsive, no detectable pulses, not breathing	Participants should start CPR with bag valve mask with a ratio of 15 compressions: 2 breaths
Participants accurately identify rhythm as ventricular tachycardia and request defibrillation	Patient is unresponsive, no detectable pulses, not breathing	Participants should deliver shock at 2 J/kg
Patient regains pulses, starts moaning	Vitals: heart rate of 160 beats per min, respiratory rate of 26 breaths per min	Move on with fluid resuscitation
	Circulation: cool extremities, capillary refill time > 4 seconds, weak pulses	
Repeat assessment after first bolus of IV normal saline or lactated ringers is finished	Vital signs: heart rate of 150 beats per min, respiratory rate of 34 breaths per min	
	Circulation: extremities remain cold, capillary refill time > 4 seconds, tachycardic, weak pulse	
Second 10 cc/kg normal saline or lactated ringer bolus hung to run over 30 minutes	Slightly more alert, pulses improved	
Facilitator prompts team leader for case summary and disposition		Team leader summarizes that this is a patient with hypovolemic shock with electrolyte abnormalities including hypernatremia and hypokalemia and requires admission for further management of electrolyte derangements

Anticipated Mistakes	
Mistake	Tips for Management
Failure to recognize that severe hypokalemia (<2.5 mEq/L) is life-threatening	A facilitator may prompt, “Do we need to worry about that potassium level?”
Not initiating cardiac monitoring or obtaining an ECG after identifying electrolyte abnormalities	A facilitator may prompt, “Are there any expected complications from these electrolyte derangements?”
Uncertainty around the volume and rate of potassium repletion	Learners can be prompted to use resources to identify the correct dose for potassium repletion

**Table 3:** Case 3 Facilitator Guide ETT, endotracheal tube; HEENT, head, ears, eyes, nose, throat; HPI, history of present illness; GU, genitourinary; PEA, pulseless electrical activity; ROSC, return of spontaneous circulation; VVR, Virtual Resus Room

Case 3 Template	
Patient information	Name: Jane; age: 12 months; weight: 9 kg; chief complaint: fever
Brief narrative description of case	Jane is presenting with five days of fever and one day of lethargy to a central hospital. On presentation, she meets the clinical definition of shock. Anticipated management includes fluid boluses, oxygen, and empiric antibiotics. The case is complicated by fluid overload resulting in increased work of breathing ultimately requiring intubation and hypotension requiring blood pressure support.
Primary learning objectives	Identify a patient in shock relying on the WHO clinical definition
	Differentiate the etiology of shock from the patient’s clinical presentation
	Identify appropriate initial therapy for a patient presenting with septic shock
	Recognize and manage complications of fluid overload
	Review steps of intubation
Critical actions	Perform initial primary survey at the start of the simulation case
	Identify that the patient meets the clinical definition of shock (cold extremities, capillary refill time > 3 seconds, weak and fast pulse)
	Create a differential for shock identifying patient presenting with likely septic shock
	Place nasal prongs to give oxygen
	Obtain IV access
	Assess for malnutrition (no evidence of wasting or peripheral edema)
	Initiate bolus of normal saline or lactated ringers per WHO shock guidelines
	Obtain and interpret emergency labs including electrolytes, glucose, and blood gas
	Administer empiric IV antibiotics
	Recognize that the patient is displaying signs of volume overload with worsening tachycardia and respiratory status
	Initiate vasopressors for hypotension
	Obtain supplies for intubation
Learner Preparation	World Health Organization Emergency Triage Assessment and Treatment Guidelines
	Virtual Resus Room participant guidelines (https://virtualresusroom.com/761-2/)

Initial Presentation	
Initial vital signs	Temperature: 38.5°C
	Pulse rate: 170 beats per minute
	Respiratory rate: 42 breaths per minute
	SpO_2_: 89%
	Blood Pressure: 70/45 mmHg
Overall appearance	Lying in mother’s arms, lethargic appearing
Actors and roles in the room at case start	Doctor #1: team leader
	Doctor #2: code recorder
	Nurse #1: medication administration
	Observer #1 (if available): critical actions checklist
	Observer #2 (if available): communication observer
	Facilitator #1: verbal simulation facilitator
	Facilitator #2: slide simulation facilitator
	Facilitator #3 (if available): parent
HPI	Jane is presenting with her parents who report she has had five days of fever and cough and one day of lethargy. They went to a local clinic and were referred urgently to the central hospital. Parents are unsure if any medications were administered. If asked for review of systems: she has had decreased appetite. No diarrhea or vomiting. She has urinated once in the last 24 hours. If asked for sick contacts: her sister has a runny nose and cough. If asked for medical history: at 6 months of age she developed a cough and difficulty breathing and was treated in a clinic. Parents are unsure what medications she received. She was not hospitalized.
Past medical/surgical history	Previously healthy
Medications/allergies/immunizations	No daily medications; no known allergies; up to date on immunizations
Family history	No family history
Social history	Lives with parents and younger sister
Physical Examination	
General	Lying on a stretcher with eyes closed, appears lethargic. Tachypneic with mild respiratory distress. Child appears well-nourished.
HEENT	Eyes are closed. Mucous membranes are moist. Oropharynx is unremarkable.
Neck	No meningismus. No lymphadenopathy.
Lungs	Tachypnea. Crackles in the right base. Breath sounds diminished in bases bilaterally. Mild subcostal retractions. Nasal flaring. No head bobbing.
Cardiovascular	Tachycardic. Normal S1 and S2. No murmurs, rubs, or gallops. Capillary refill 4 seconds, weak pulse. Cool extremities
Abdomen	Abdomen is soft and non-tender. No rebound or guarding. Bowel sounds active.
Neurological	Eyes are closed though open to voice and stimuli. Moves all extremities.
Skin	Extremities are cold to touch. Pale. No rash.
GU	Normal external female genitalia.

Instructor Notes - Changes and Case Branch Points		
Intervention/Time Point	Change in Case	Additional Information
Perform primary survey to assess airway, breath sounds, and circulation	Initial vital signs: temperature of 38.5°C, pulse rate of 170 beats per minute, respiratory rate of 42 breaths per minute, blood pressure of 70/45 mmHg, pulse oximetry of 89%	Given the patient’s circulatory status and examination, she meets the clinical definition of shock
	Airway: intact, patient moaning	
	Breath sounds: breath sounds are coarse throughout and diminished at the bases, mild retractions.	
	Circulation: cold extremities, capillary refill time > 4 seconds, tachycardic, weak pulse	
	Disability/neurologic assessment: lethargic on stretcher, opens eyes to voice	
History is gathered from the parent to determine the likely etiology of shock presentation	Parent describes history of fever and cough	Team leader states that history is most consistent with septic shock in the setting of a lower respiratory infection
Patient is assessed for malnutrition	No visible wasting, no peripheral edema	
Nasal cannula is placed on patient (1-2 liters/minute with goal oxygen saturation > 90%)	Oxygen saturation improves to 92%	
IV access is requested (or could utilize IV placed at outside clinic)	IV placed	
20 cc/kg normal saline or lactated ringer bolus is hung to be run over 30 minutes	No change	If participants give fluids too quickly, advance to the portion of the simulation where the patient decompensates following the second bolus
Empiric antibiotics are administered (ceftriaxone 75 mg/kg IV)	No change	
Participants may ask for chest X-ray	No change	Facilitator states that X-ray is currently unavailable and films will be delayed
Labs including electrolytes and glucose are requested	No change	After the labs are ordered, the facilitator states that 30 minutes have passed and the first bolus is completed. Advance VRR clock by 30 minutes. Labs result as follows: sodium of 133 mEq/L, potassium of 3.5 mEql/L, chloride of 105 mEq/L, bicarbonate of 15 mEq/L, glucose of 60 mg/dL (3.3 mmol/L)
Repeat assessment after the first bolus is administered	Repeat vital signs: heart rate of 170 beats per min, Respiratory rate of 42 breaths per min, pulse oximetry of 92%, Blood pressure of 80/50 mmHg	
	Airway: intact	
	Breath sounds: breath sounds remain coarse throughout	
	Circulation: extremities remain cold, capillary refill time > 4 seconds, tachycardic, weak pulse	
	Disability/neurologic assessment: awake	
Second 10 cc/kg normal saline or lactated ringer bolus hung to run over 30 minutes	No immediate change	If participants do not order a second bolus, the facilitator states “A bedside nurse is asking about giving the patient more fluids.”
		After the second bolus has been ordered, facilitator to state that 30 minutes have elapsed and the bolus is complete. Advance the VRR time clock by 30 minutes.
Patient develops worsening tachypnea and tachycardia following the second fluid bolus	Repeat vital signs: heart rate of 168 beats per min, Respiratory rate of 58 breaths per min, blood pressure of 60/40 mmHg, pulse oximetry of 85%	
	Airway: intact	
	Breath sounds: breath sounds are coarse, increasingly tachypneic	
	Circulation: extremities remain cold, capillary refill time > 4 seconds, tachycardic, weak pulse	
	Disability/neurologic assessment: patient appears more lethargic	
Increased respiratory support requested	If oxygen by nasal cannula is increased, no change	If learners ask for blood gas: pH 7.1, pCO_2_ 63, HCO_3_ 12, base deficit 10, lactate 8
	If a non-rebreather mask is placed, increase saturation to 89% with no change in respiratory distress	
	If intubation is requested, facilitator to state “the ICU nurse is asking if it is safe to proceed with intubation with his current blood pressure?”	
Vasopressors are requested: adrenaline/epinephrine drip (0.05 mcg/kg/min) or epinephrine boluses	Repeat vital signs: heart rate of 160 beats per minute, blood pressure of 80/55 mmHg, respiratory rate of 60 breaths per minute, Pulse oximetry: 89-91%	Learners to proceed with rapid sequence intubation
	Perfusion improves, extremities warmer	
If vasopressors are not requested before rapid sequence intubation	Patient to go into PEA arrest and after two doses of epinephrine will obtain ROSC	
Supplies are obtained for rapid sequence intubation		ETT: Diameter of cuffed ETT = (age/4) + 3.5
		Suction catheter size = 2 x ETT
		Laryngoscope with blades
		20 mL syringe
		Induction agent (ketamine 1 mg/kg)
		Paralytic (succinylcholine 1.5 mg/kg)
Repeat vital signs requested after intubation	Vital signs post-intubation: heart rate of 150 beats per minute, respiratory rate ventilated, SpO_2_ of 99%, blood pressure of 80/55 mmHg	Facilitator stops the scenario once learners have stabilized the blood pressure and the patient is intubated
	Airway: successfully intubated	
	Breath sounds: clear bilateral ventilated breath sounds	
	Circulation: extremities warmer, capillary refill 3 seconds	
	Disability/neurologic assessment: sedated	
Patient is signed out to the pediatric intensive care unit		Example summary statement: 12-month-old, 9 kg infant presenting with septic shock, two IV fluid boluses were administered followed by empiric antibiotics, vasopressor support, and intubation for worsening respiratory status

Anticipated Mistakes	
Mistake	Tips for Management
Continuing to administer IV fluids after the patient develops worsening tachypnea	It is unclear if the patient received additional IV fluids at the outside clinic prior to arrival. Patients in septic shock are at risk of fluid overload and pulmonary edema and the patient’s development of tachypnea and desaturation are suggestive of this. The patient’s hypotension should be supported with vasopressors instead of additional fluid.
Failure to initiate blood pressure support prior to intubation	If the learner requests rapid sequence intubation without addressing the patient’s hypotension, the facilitator is to prompt with “a nurse is asking if it is safe to intubate with his blood pressure.” If the learner suggests additional fluids for blood pressure support the facilitator can state “she has already received 2 boluses, are there any concerns with additional fluids?”

For prebriefing, the facilitator guides were distributed to each of the case facilitators via email. Facilitators reviewed the scenarios and divided facilitator roles including management of the slides (i.e., updating vital signs), responding to learners’ prompts (utilizing the case notes with branching points provided in the guide), and playing the role of the patient’s parent for additional history. Learner participants were provided with a link for the VRR participant guidelines (https://virtualresusroom.com/761-2/) to familiarize them with the platform prior to the simulation [5].

In case 1, the patient is presenting to a rural clinic setting with history of diarrhea and lethargy. The ideal flow scenario is as follows: The learners enter the room to find the patient appearing lethargic and severely dehydrated. On initial assessment, they note that he has impaired circulation and meets the clinical definition of shock with delayed capillary refill greater than 3 seconds, cold extremities, and a weak and fast pulse. From the clinical history and physical examination, it is suspected that the patient has hypovolemic shock. Intravenous access is promptly obtained and the patient is given a bolus of normal saline or lactated ringers. If point-of-care electrolytes or glucose are requested, they are informed that this testing is not available at this facility. Glucose should thus be administered empirically. After glucose and the initial bolus of fluids, the patient is reassessed with minimal improvement. The patient is given an additional bolus of crystalloid fluid. The patient has minimal improvement in his vitals and examination. Given the lack of intensive care resources and laboratory evaluation, it is recommended that the patient be transferred to a referral or district-level hospital for additional management.

**Figure 1:**
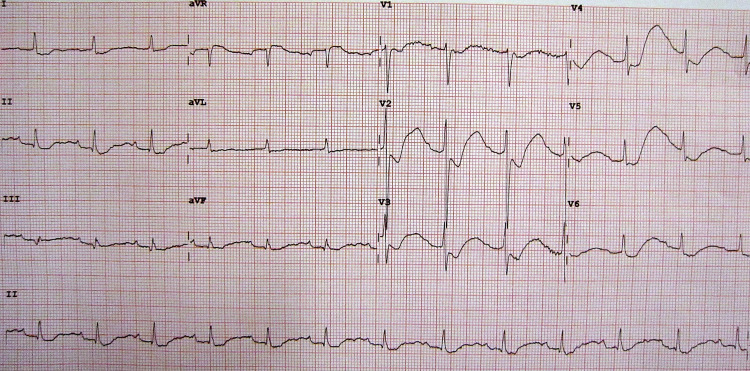
Hypokalemia ECG Author James Heilman, MD: Creative Commons Attribution-Share Alike 3.0 Unported license. Available at https://commons.wikimedia.org/wiki/File:LowKECG.JPG

In case 2, the patient is again presenting with diarrhea and lethargy, though this time to a district-level hospital. The ideal flow scenario is as follows: The learners enter the room to find the patient lethargic and severely dehydrated. On initial assessment, they note that he has impaired circulation and meets the clinical definition of shock with delayed capillary refill greater than 3 seconds, cold extremities, and a weak and fast pulse. From the clinical history and physical examination, it is suspected that the patient has hypovolemic shock. Intravenous access is promptly obtained, and the patient is given a bolus of normal saline or lactated ringers. Emergency labs are obtained including electrolytes and glucose. The labs are remarkable for hypernatremia and marked hypokalemia. An EKG is obtained revealing T-wave flattening and visible U wave (Figure 1). If potassium supplementation is not initiated, the patient becomes unresponsive with pulseless ventricular tachycardia. The participants should initiate CPR and deliver a shock via the defibrillator. The patient’s vital signs stabilize, and a second fluid bolus is administered. Decision is made to admit for further management and monitoring of electrolyte derangements.

In case 3, the patient is presenting with lethargy and fever to a district-level hospital. The ideal flow scenario is as follows: The learners enter the room to find the patient lethargic. On initial assessment, they note that she has impaired circulation and meets the clinical definition of shock per WHO with delayed capillary refill greater than 3 seconds, cold extremities, and a weak and fast pulse. They additionally note she is febrile with signs of increased work of breathing. From the clinical history and physical examination, it is suspected that the patient has septic shock in the setting of a lower respiratory tract infection. Intravenous access is promptly obtained, and the patient is given a bolus of normal saline or lactated ringers. She is started on supplemental oxygen, and emergency labs are obtained including electrolytes and glucose. She is additionally given empiric antibiotics. On repeat evaluation, she has had minimal improvement and is given a second fluid bolus. While the second bolus is being administered, she develops worsening tachypnea concerning for pulmonary edema. The patient does not have improvement with higher flow through the nasal cannula or with the non-rebreather mask. Additionally, the patient becomes hypotensive and requires blood pressure support with vasopressors prior to intubation. The learner intubates the patient and her vitals improve.

Supplementary materials 

**Figure 2:**
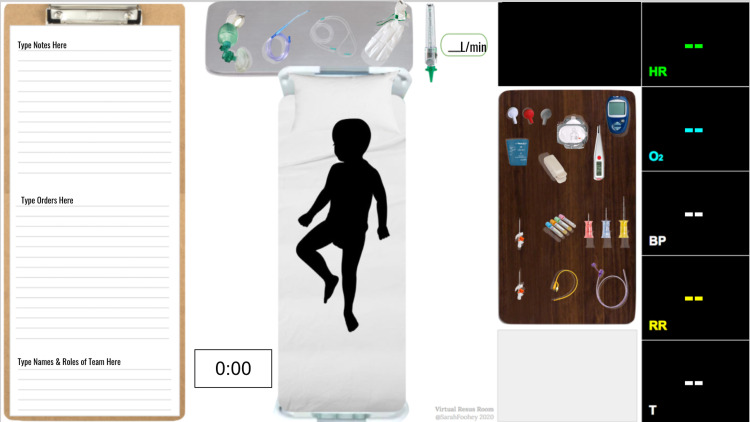
Virtual Resuscitation Room https://virtualresusroom.com/

**Figure 3:**
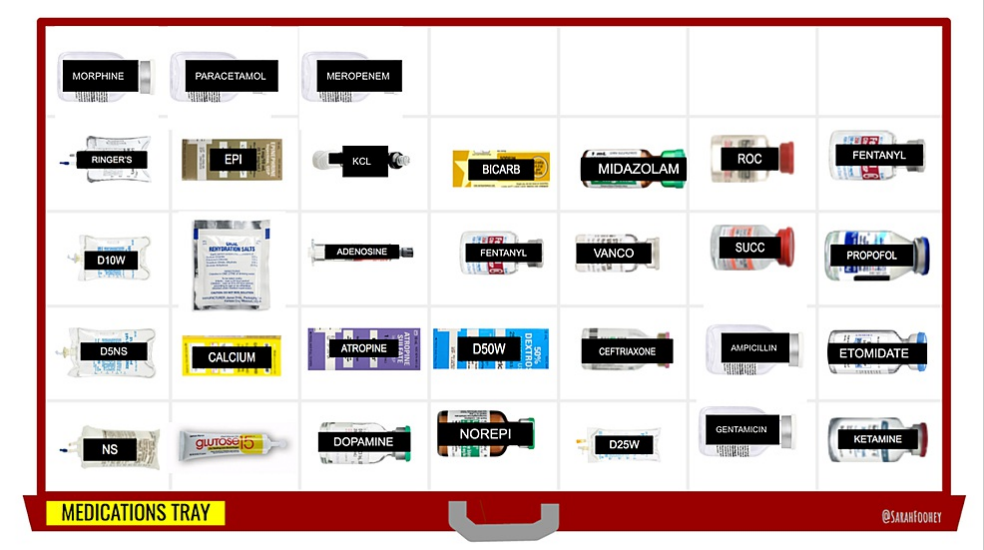
Medication Tray https://virtualresusroom.com/

**Figure 4:**
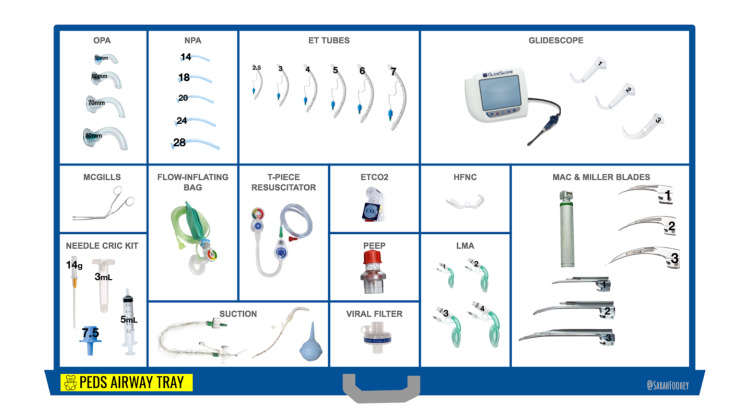
Pediatric Airway Tray https://virtualresusroom.com/

VRR Google Slides decks were developed for each of the three case scenarios using the open-access template materials available on the VRR website [5]. The figures are screen-captures from the slide decks showing the virtual resuscitation room (Figure 2), the medication tray (Figure 3), and the pediatric airway tray (Figure 4). These slides were accessed and edited simultaneously by the learners and facilitators to reflect real-time changes in the case. Medications and airway supplies were copied and pasted into the primary resuscitation room when required or administered.

Feedback and debriefing 

**Figure 5:**
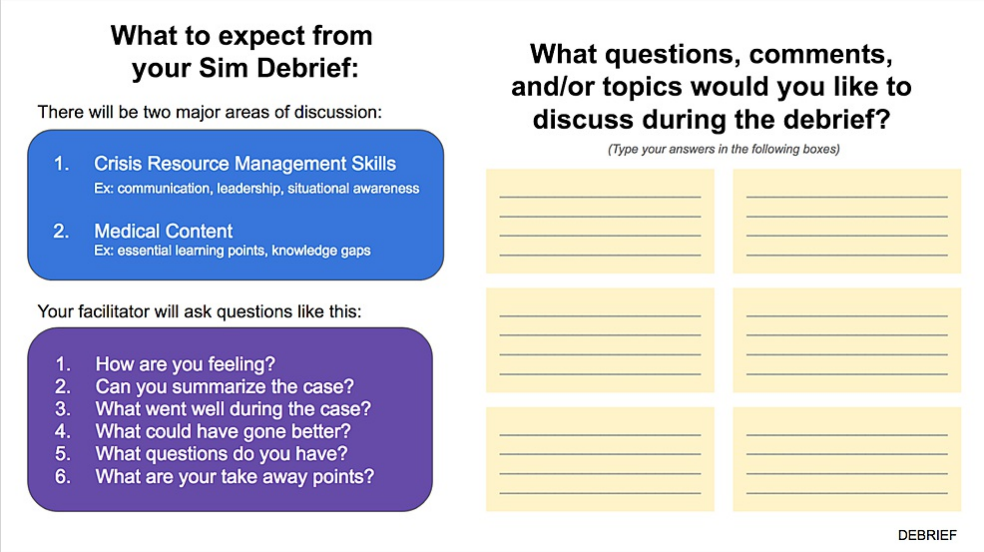
Debriefing Slide

The simulations were completed with seven UW PEM fellows, followed by five PECC-Kenya fellows, for a total of 12 initial participants. The time needed to complete all three scenarios required two separate sessions of 60-90 minutes each. Seven PECC-Kenya fellows participated in an additional session. Debriefing was carried out at the end of each case via a slide prompting discussion (Figure 5). Two major topics were discussed during debriefing: crisis resource management skills (communication, leadership) and medical content (essential learning points, knowledge gaps). Key learning points were summarized at the end of each case.

Participants were additionally emailed a link to an evaluation in REDCap, with results summarized in Table 4. This electronic survey asked participants to rate their confidence with the learning objectives before and after the session on a five-point Likert scale (very confident, confident, neutral, unconfident, very unconfident).

**Table 4:** Post-simulation Feedback Survey Responses

Statement	Confident or Very Confident (Before Simulation) (n = 8)	Confident or Very Confident (After Simulation) (n = 8)
Demonstrate ability to assess and emergency manage a patient presenting in shock	87%	100%
Understand how fluid resuscitation differs in areas with variable resources and access to care	25%	87%

Statement	Agree or Strongly Agree (n = 8)
Prior to this, I have participated in telesimulation in the past	50%
The cases utilized during this simulation are relevant to work in resource-limited global health settings	100%
The simulation cases were effective in teaching basic fluid resuscitation skills in settings with variable resources	100%
The scenarios allowed practice of effective teamwork and communication skills	83%
Telesimulation is an effective medium for learning	100%

Participants overall felt that virtual simulation was an effective medium for learning. VRR was new for 50% of the fellows and they expressed desire for continued practice with the format. Verbal feedback revealed that VRR worked best for participants with video capability and a computer-based reliable internet connection.

## Discussion

The use of virtual simulation provided an effective modality for education on recognition and management of hypovolemic and septic shock, utilizing international guidelines. The VRR platform was well received by the PEM and PCCM fellows in Kenya. The cases were designed to increase in medical complexity, which allowed time for fellows to adapt to the modality. Utilizing the orientation video available on the VRR website assisted in preparing learners for the simulation format. Allowing the slides to be easily customizable provided a more realistic scenario for settings with variable resources. To make future simulation scenarios more realistic for providers practicing in low- to middle-income countries, simulations can be designed to involve fewer active participants, as these physicians expressed that they typically are functioning in multiple roles in a resuscitation. Expanding the number of participants observing the simulation, including observing communication and utilizing a critical actions checklist, would continue to allow for multiple learners to participate in the simulation. While survey results demonstrated improved self-confidence in the management of shock, further research, utilizing direct comparison of virtual simulation education to traditional in-person simulation, could further validate the efficacy of this modality, as has been demonstrated in prior virtual simulation studies [3-4].

## Conclusions

VRR facilitated international collaboration and allowed for effective remote simulation education. Designing virtual simulation scenarios with content specifically highlighting management differences in settings with different resources allows for an adaptable and more realistic learning environment for providers practicing in or traveling to low- to middle-income countries. Further implementation is planned to assess VRR effectiveness and usability in multiple global settings.

## References

[1] Davis D, Warrington SJ (2021). Simulation training and skill assessment in emergency medicine. StatPearls [Internet].

[2] McCoy CE, Sayegh J, Alrabah R, Yarris LM (2017). Telesimulation: an innovative tool for health professions education. AEM Educ Train.

[3] Umoren R, Bucher S, Hippe DS (2021). eHBB: a randomised controlled trial of virtual reality or video for neonatal resuscitation refresher training in healthcare workers in resource-scarce settings. BMJ Open.

[4] Nas J, Thannhauser J, Vart P (2020). Effect of face-to-face vs virtual reality training on cardiopulmonary resuscitation quality: a randomized clinical trial. JAMA Cardiol.

[5] (2021). Virtual Resus Room. https://virtualresusroom.com/.

[6] (2021). Children: improving survival and well-being. World Health Organization.

[7] Maitland K, Kiguli S, Opoka RO (2011). Mortality after fluid bolus in African children with severe infection. N Engl J Med.

[8] World Health Organization (2021). Updated guideline: paediatric emergency triage, assessment and treatment: care of critically-ill children. https://apps.who.int/iris/handle/10665/204463.

[9] Weiss SL, Peters MJ, Alhazzani W (2020). Surviving Sepsis Campaign International Guidelines for the management of septic shock and sepsis-associated organ dysfunction in children. Pediatr Crit Care Med.

[10] Wiseman J, Snell L (2008). The deteriorating patient: a realistic but ‘low-tech’ simulation of emergency decision-making. Clin Teach.

